# Renal Preservation During Open Abdominal Aortic Aneurysm (AAA) Repair in a Patient With a Pelvic Kidney: A Case Report and Surgical Insight

**DOI:** 10.7759/cureus.87428

**Published:** 2025-07-07

**Authors:** Abdulkreem Al-Juhani, Sultan Khoja, Abdullah Abdullah, Rodan Desoky

**Affiliations:** 1 General Surgery, King Abdulaziz University, Faculty of Medicine, Jeddah, SAU; 2 Vascular Surgery, King Abdulaziz University Hospital, Jeddah, SAU; 3 Medicine, Alfaisal University College of Medicine, Riyadh, SAU

**Keywords:** abdominal aortic aneurysm, aberrant renal artery, anatomical variation, ectopic kidney, open aaa repair, renal function recovery, renal perfusion, vascular surgery

## Abstract

Repair of an abdominal aortic aneurysm (AAA) becomes significantly more complex when complicated by the presence of atypical renal vasculature and an ectopic kidney, as these anatomic variations pose distinct surgical challenges. In these cases, the surgeon must not only address the aneurysmal part of the aorta but also manage the aberrant renal anatomy to ensure the preservation of kidney function. This is critical in open AAA repair, where prolonged aortic clamping and manipulation near aberrant renal vessels can compromise renal perfusion. Preservation of renal function under these circumstances is technically demanding but essential for a successful outcome. This case report presents a 59-year-old male patient with an infrarenal abdominal aortic aneurysm and a pelvic ectopic kidney, in which the renal artery originated anomalously from the distal aorta. The patient underwent successful open surgical repair with a comprehensive planning and intraoperative strategy tailored to protect renal function while treating the aneurysm, emphasizing the importance of individualized surgical approaches in managing such rare and anatomically complex presentations.

## Introduction

Abdominal aortic aneurysm (AAA) is a common vascular disease that is often found by accident in older people. Standard repair methods, whether open surgery or endovascular, are well known. However, anatomical differences such as ectopic kidneys or aberrant renal arteries make surgery and clinical work much harder. Approximately one in 900 people have ectopic pelvic kidneys, which generally have unusual arterial supplies. This makes aortic cross-clamping more challenging and increases the risk of renal ischemia after AAA repair [1].

 In these situations, maintaining kidney function is crucial for optimal outcomes after surgery. Aberrant renal arteries, especially those originating from the aneurysmal sac or the distal aorta, require careful planning before surgery, accurate identification during surgery, and often revascularization to maintain kidney function properly [2]. Poor management can cause acute kidney injury, which requires dialysis, or it can cause permanent kidney loss [3].

Therefore, it is essential to have a personalized surgical plan that takes into account any potential issues with the blood vessels. Recent research shows that cool renal perfusion, vascular management via vessel loops, and selective reimplantation of renal arteries are all good ways to keep the kidneys healthy during open repair of complex AAAs [4]. These methods are especially critical for individuals who have only one kidney or an ectopic kidney, where renal reserve is low. Long-term results also demonstrate that renal artery reimplantation is a viable and safe option when performed carefully during open repair, providing better blood flow than ligation or embolization [5].

This case report discusses the successful open surgical repair of a patient with a right ectopic pelvic kidney and an infrarenal AAA. This case illustrates the technical challenges associated with complex renal vasculature and the clinical effectiveness of intraoperative renal protection, evidenced by a reduction in blood creatinine levels from 140 µmol/L preoperatively to 78 µmol/L postoperatively. This case illustrates the necessity of individualized surgical planning to achieve favorable outcomes in complex vascular procedures due to anatomical considerations.

## Case presentation

Patient profile

A 59-year-old male with a 5.6 cm infrarenal AAA and an ectopic right pelvic kidney was being evaluated for elective repair of his aortic aneurysm. His AAA was discovered incidentally after workup for left kidney stones two years prior. At that time, his aneurysm was small, measuring 3.5 cm in maximum diameter. Additionally, he was found to have a functioning ectopic right pelvic kidney. His aneurysm was monitored, and surprisingly, over the course of two years, he had rapid expansion of his aneurysm over the surveillance period, which measured 5.6 cm in maximum diameter on CT scan. His ectopic right pelvic kidney was supplied by a single aberrant right renal artery that originated from his aorta anteriorly near the aortic bifurcation. His left kidney and left renal artery were in their normal anatomic location.

Regarding his past medical history, he has been diagnosed with diabetes mellitus (DM), hypertension (HTN), and dyslipidemia. He had mild renal insufficiency with elevated creatinine in the range of 120-140 µmol/L. He has no family history of AAA, aortic pathology, or history of renal anomalies. He quit cigarette smoking after the initial diagnosis of his AAA two years ago.

Upon assessment, he had no syndromic features or any signs suggestive of connective tissue disease. His aneurysm was palpable on abdominal examination and was non-tender. A vascular examination of the lower limbs was normal, with palpable bilateral femoral, popliteal, posterior tibial, and dorsalis pedis artery pulses.

Given the size of his AAA along with the rapid expansion, he was recommended for elective repair. Due to his anomalous right renal artery, a standard endovascular repair (EVAR) was not possible without sacrificing his ectopic right kidney. His options for repair were either open, a hybrid, or an advanced complex EVAR. As he was relatively young and fit for open surgery, we recommended open repair to preserve his right kidney and give him the most durable repair. Informed written consent was obtained from the patient for open-access publication of this case report.

Perioperative assessment

Table 1 presents the perioperative laboratory investigations.

**Table 1:** Perioperative laboratory investigation values WBC: white blood cell; ALT: alanine aminotransferase; AST: aspartate aminotransferase; INR: international normalized ratio; PT: prothrombin time; aPTT: activated partial thromboplastin time

Parameters	Preoperative	Postoperative Day 3	Reference Ranges
Hemoglobin (g/dL)	13.2	13.8	13.5–17.5 g/dL
WBC (×10⁹/L)	7.8	7.4	4.5–11.0 ×10⁹/L
Platelets (×10⁹/L)	256	248	150–400 ×10⁹/L
Creatinine (µmol/L)	140	78	53–106 µmol/L
Urea (mmol/L)	5.1	4.6	2.5–6.4 mmol/L
ALT (U/L)	24	21	10–40 U/L
AST (U/L)	19	17	12–38 U/L
INR	1	1	11–15 sec
PT (sec)	12.5	12.2	0.9–1.2 sec
aPTT (sec)	29	28	25–40 sec
Sodium (mmol/L)	138	139	136–146 mmol/L
Potassium (mmol/L)	4.3	4.2	3.5–5.0 mmol/L
Chloride (mmol/L)	102	101	95–105 mmol/L
Bicarbonate (mmol/L)	23	24	22–28 mmol/L

Surgical procedure

The surgery was conducted using a standard transperitoneal approach through a midline laparotomy under general anesthesia. Upon entering the abdominal cavity, the ectopic right pelvic kidney was easily identified and palpable in the right lower quadrant. The aorta was approached in the standard fashion, after mobilizing the small bowel and exposing the retroperitoneum. The retroperitoneum and ligament of Treitz overlying the abdominal aorta were dissected. The left renal vein was identified, which was in its normal position. A proximal clamp site was developed at the long neck below the left renal artery. Distally, the aortoiliac segment was dissected carefully. The origin of the aberrant right renal artery was easily identified, which originated anteriorly at the level of the aortic bifurcation. Dissection and control of the right renal artery were not challenging. Following control of the right renal artery, both common iliac arteries were dissected and controlled. Once clamp sites and control were established, the patient was systemically heparinized, and IV mannitol was administered for renal protection. The sequence of clamping was as follows: first, the right renal artery; then, both common iliac arteries distally; and finally, proximal clamping.

The AAA was incised, and back-bleeding lumbers were oversewn. A small cannula was inserted into the right renal artery orifice and continuously flushed with cold saline as an adjunct for renal protection. A 16x8 silver-impregnated Dacron graft was used for the aortic reconstruction. Upon completion of the proximal aortic anastomosis, the aberrant right renal artery was reimplanted into the right limb of the graft. This was achieved by making a longitudinal graftotomy and performing an end-to-side anastomosis of the right renal artery using 4-0 Prolene sutures. Once the anastomosis was done, the graft was flushed, and the right kidney was reperfused. Interestingly, the origin of the transacted right renal artery had some atherosclerotic plaque causing narrowing, which was endarterectomized prior to the anastomosis.

**Figure 1:**
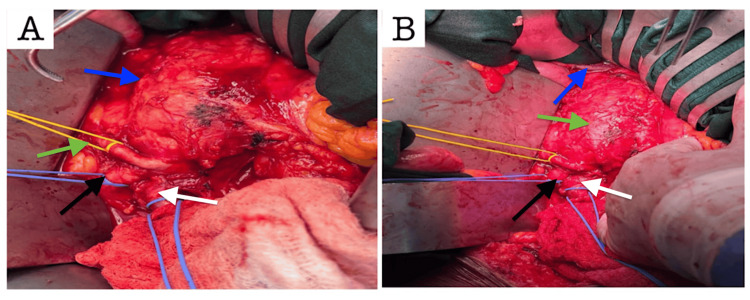
The abnormal right renal artery and aneurysmal infra-renal aorta are visible in the intraoperative dissection (A). The abnormal renal artery and left renal vein are identified, emphasizing important anatomical features for revascularization planning (B). (A) Blue arrow: aneurysmal Infrarenal aorta; green arrow: aberrant right renal artery; black arrow: right common iliac artery; white arrow: left common iliac artery (B) Blue arrow: left renal vein; green arrow: aneurysmal infrarenal aorta; black arrow: right common iliac artery; white arrow: left common iliac artery

**Figure 2:**
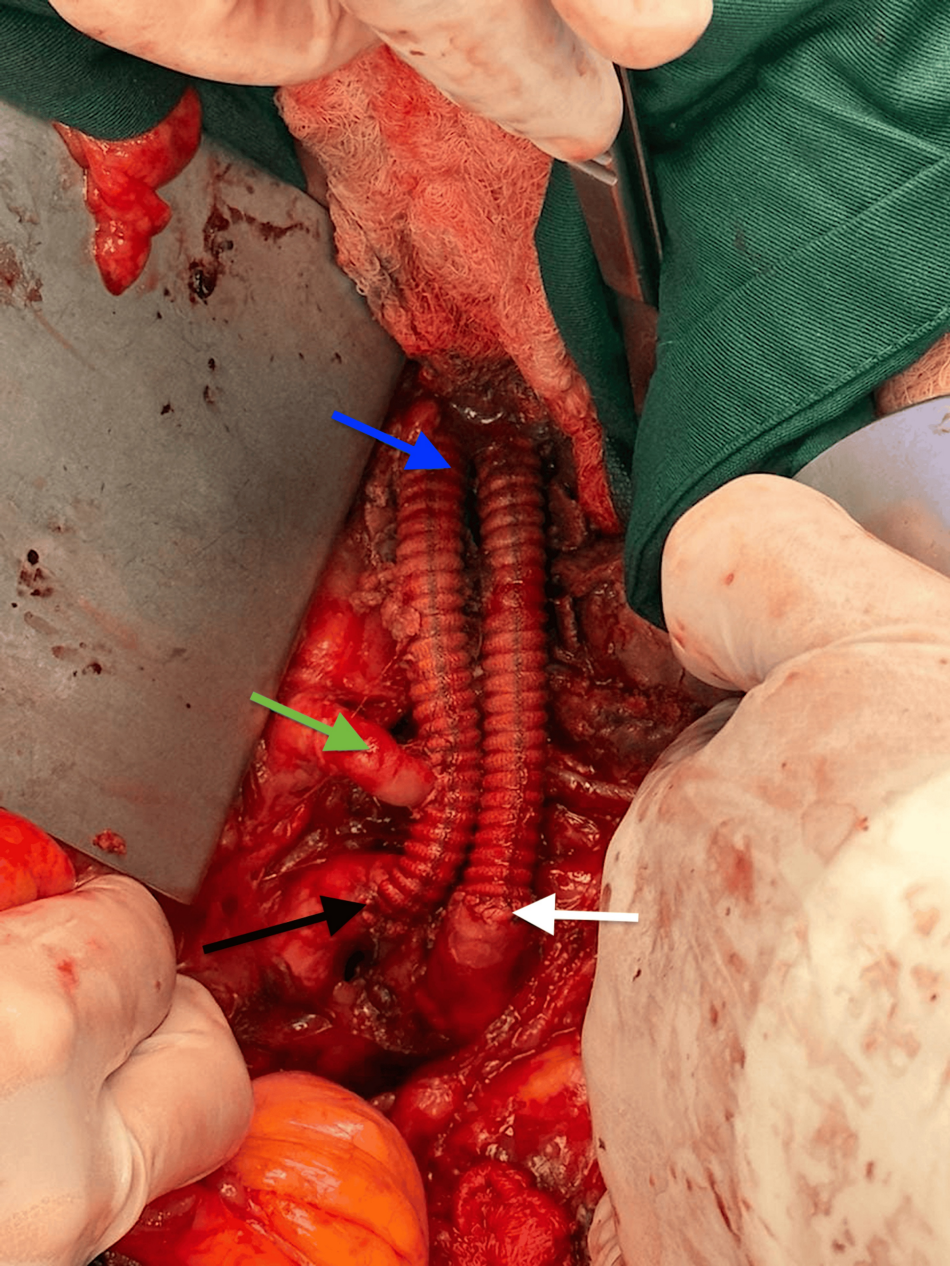
End-to-end anastomoses to the iliac arteries, reimplantation of the aberrant renal artery, and a bifurcated Dacron graft comprise the final reconstruction. Blue arrow: aorto-bi-iliac repair using a 16x8 bifurcated Dacron graft; green arrow: end-to-side reimplantation of the aberrant right renal artery into the right iliac limb; black arrow: right common iliac artery with end-to-end anastomosis; white arrow: left common iliac artery with end-to-end anastomosis

The rest of the procedure was completed in the standard open AAA repair fashion, as illustrated in Figures 1, 2. Both limbs of the graft were anastomosed to the corresponding common iliac arteries in an end-to-end anastomosis. Once hemostasis was achieved, the sac and the retroperitoneum were closed, followed by the closure of the abdominal wall. The estimated blood loss was under 500 mL. A cell saver was used throughout the procedure. The procedure was well tolerated by the patient, and no intraoperative complications occurred. He was extubated in the operating room and transferred to the ICU in a stable condition for monitoring as planned.

The patient's postoperative course was quite unremarkable. He was transferred from the ICU to the regular surgical ward on postoperative day one. He was discharged home in good condition on postoperative day five after regaining normal bowel function and adequate pain control.

Surprisingly, the patient's serum creatinine has improved and normalized on postoperative day one compared to his baseline. Throughout his postoperative hospital stay, he maintained normal renal function with a consistent serum creatinine level in the 70s µmol/L range, which remained constant at the patient's two-week follow-up.

## Discussion

The management of an AAA alongside an ectopic kidney and aberrant renal vasculature is an uncommon yet significant difficulty in vascular surgery. Ectopic kidneys occur in approximately one in 900 births and are often located in the pelvis. These kidneys generally exhibit anomalous arterial supply, frequently originating from the distal aorta, iliac arteries, or the inferior mesenteric artery, and lack a distinct renal pedicle, hence complicating any operation involving the abdominal aorta [6].

The anomalous renal artery originated directly from the aneurysmal sac, making it susceptible to disruption during conventional aneurysm surgery. The preservation of renal function depended on the surgeon's capacity to locate, isolate, perfuse, and reimplant this conduit. Neglecting to address such an artery, particularly in individuals with solitary or ectopic kidneys, may result in renal infarction, acute kidney damage (AKI), and potentially long-term need for dialysis [7].

The surgical approach employed involved cold renal perfusion and the prompt reimplantation of the anomalous renal artery into a bifurcated Dacron graft. Cold perfusion mitigates ischemic damage during cross-clamping and is particularly advantageous when the duration of renal warm ischemia exceeds 30 minutes. Numerous investigations endorse this methodology; for example, Krzyzaniak et al. (2025) noted markedly reduced postoperative creatinine levels in individuals subjected to cold perfusion compared to those treated only with clamping during intricate aortic procedures [8].

The renal artery was reimplanted instead of ligated due to intraoperative observations and preoperative imaging that verified it as the exclusive supply to the pelvic kidney. The Society for Vascular Surgery recommends the preservation or revascularization of any auxiliary or aberrant renal artery that supplies above 25-30% of renal parenchyma, particularly in cases of ectopic or solitary kidneys [9]. A multicenter registry analysis revealed that patients who underwent renal artery reimplantation experienced markedly improved renal outcomes and a reduction in postoperative dialysis rates compared to those who received ligation or embolization [10].

Although EVAR is commonly utilized for standard AAA management, it is often contraindicated or less feasible in anatomically challenging scenarios such as this. Anomalous renal arteries originating from the aneurysmal sac impede the secure insertion of standard stent grafts without compromising renal perfusion. While fenestrated and branched EVAR systems have expanded treatment options, they require careful design and custom graft fabrication and are rarely suitable for pelvic kidneys due to their irregular vascular anatomy. A recent meta-analysis by Lee et al. (2023) indicates that open repair is the superior method for AAA with concurrent renal problems, exhibiting improved long-term renal outcomes and procedural effectiveness [11]. The patient's renal function significantly enhanced after surgery, with blood creatinine levels decreasing from 140 µmol/L preoperatively to 78 µmol/L on the third postoperative day. This rapid normalization aligns with the findings of Soto et al. (2025), which demonstrated that early postoperative renal recovery is substantially associated with intraoperative cold perfusion and the mitigation of prolonged renal ischemia [12]. Moreover, the consistent implementation of perioperative techniques, including the avoidance of nephrotoxic agents, adjustment of fluid balance, and prompt movement, likely facilitated this positive recovery. This case underscores the importance of preoperative imaging using contrast-enhanced computed tomography angiography (CTA) to detect kidney abnormalities and formulate a surgical plan. Intraoperative instruments, such as Doppler ultrasound or back-bleeding evaluation of the renal arteries, can assist in verifying the functional importance of anomalous vasculature and ensuring sufficient revascularization [13].

The effective handling in this instance underscores that, with suitable preoperative evaluation, intraoperative strategizing, and renoprotective measures, open AAA repair can be conducted safely despite the intricate renal vascular anatomy. This method is especially crucial in resource-constrained environments where sophisticated EVAR platforms may be unavailable or impractical to use.

## Conclusions

Performing open AAA repair in individuals with an ectopic kidney and aberrant renal artery is both feasible and safe when supported by meticulous preoperative imaging and intraoperative renal safeguarding. In this case, the use of cold perfusion and prompt reimplantation of the aberrant artery successfully maintained renal function, as evidenced by the swift normalization of serum creatinine and the timely postoperative recovery. This instance underscores the significance of personalized surgical planning, especially for patients with anatomically intricate vascular variants, which can lead to favorable surgical outcomes even in such complex vascular presentations. The case also highlights that pursuing open repair surgery, rather than endovascular options, was the necessary approach for the preservation of the functional kidney.
